# ‘But there are so many referrals which are totally … only generating work and irritation’: a qualitative study of physicians’ and nurses’ experiences of work tasks in primary care in Sweden

**DOI:** 10.1080/02813432.2022.2139447

**Published:** 2022-11-03

**Authors:** Eva Anskär, Magnus Falk, Annette Sverker

**Affiliations:** a Department of Health, Medicine and Caring Sciences, Primary Health Care Center Cityhälsan Centrum, Linköping University, Linköping, Sweden; b Department of Health, Medicine and Caring Sciences, Kärna Primary Health Care Center, Linköping University, Linköping, Sweden; c Department of Activity and Health, Linköping University, Linköping, Sweden; d Department of Health, Medicine and Caring Sciences, Linköping University, Linköping, Sweden

**Keywords:** Primary care, legitimacy of work tasks, competence, working situation, qualitative study

## Abstract

**Objective:**

This study explored the perceptions of physicians and nurses in Swedish primary care regarding the legitimacy of their work tasks and the use of their professional competence.

**Design and setting:**

This qualitative study was based on manifest content analysis. Data were collected with individual semi-structured interviews of physicians and nurses at publicly managed primary care centres in the Region Östergötland, Sweden. To include both large and small primary care centres, we applied strategic sampling. Among 15 primary care centres invited, nine agreed to participate, including four urban, two suburban, and three rural centres.

**Subjects:**

The study included 11 physicians and 13 nurses from nine primary care centres.

**Main outcome measures and results:**

The physicians and nurses perceived several of their work tasks as illegitimate. In addition, they experienced work-related difficulties, due to resource shortages, challenging electronic data systems, work inefficiencies, and that there were insufficient cooperation with, and problems drawing the line between, primary care and hospital care responsibilities. However, most found that their individual expertise was appropriately used, overall.

**Conclusions:**

Strained work situations and illegitimate work tasks may inflate discontentment and lead to negative work stress. Nevertheless, the staff felt that competence was used appropriately in the tasks considered legitimate.

**Key Points**
- Physicians and nurses in primary care in Sweden described several work tasks as illegitimate.
- Physicians and nurses described problems with heavy workloads, resource shortages, electronic data-system challenges, inefficiencies and with cooperation and drawing the line between primary care and hospital care.
- Overall, physicians and nurses believed their individual expertise was used appropriately.
- To uphold sustainable working conditions and thoughtful use of staff competence, organisational measures, such as redistributing staff from hospital to primary care were proposed.

## Introduction

Healthcare centres should use resources efficiently, particularly human resources, which are limited [1,2]. In public organisations (including healthcare), healthcare staff spend a considerable amount of time performing administrative tasks, in addition to their professional work tasks (i.e. work tasks they were trained to perform) [1]. These administrative work tasks include completing electronic health records, scheduling appointments, managing referrals, entering data into quality registries, attending meetings, preparing work schedules and managing communications, such as e-mail [3]. In Sweden, primary care staff spend as little as 37% of their work time directly with patients. This circumstance might affect their perceptions of the legitimacy of their work tasks [3]. In this context, legitimate work tasks are tasks that workers perceive as meaningful and that they expect to perform in a specific profession or person [4]. One Swedish study found that physicians had a more negative perception of their psychosocial work environment compared to all other professionals in primary care centres [3]. The trust given to healthcare workers in performing a job most likely plays an important role [5] in whether these workers find meaning in their work. For primary care staff, meaningful work – i.e. legitimate work tasks – is associated with the potential of achieving optimal clinical results [6,7]. However, the perception of an illegitimate task probably also depends on the context of the work and individual factors. Consequently, these perceptions might be different among primary care workers in Sweden, compared to, for example the US, or even Norway and Denmark.

Competence is more than possessing professional knowledge and skills; the interplay between the individual and the clinical setting is also very important [7]. In addition, good communication, collaboration, management and scholarship are needed to exercise professional competence to the fullest [7]. In Sweden, the title ‘registered nurse’ is used to mean a licensed nurse with an advanced education [8], which requires at least three years, and often four years or more, of study. Halcomb and Ashley pointed out the importance of encouraging nurses to use the full extent of their unique competence and skills [9].

Despite the known negative influence of illegitimate work tasks, they are common [4,10,11]. Semmer et al. categorised illegitimate work tasks as follows: (i) tasks perceived as unnecessary for the general performance of duties or tasks that would be unnecessary in a more organised environment; and (ii) tasks perceived as unreasonable, and therefore, should not be performed by a specific individual or professional group [4,10,11]. The perception of tasks as illegitimate is often associated with counterproductive work behaviour, such as poor relations with colleagues and using the internet for matters unrelated to work [4]. Work that includes a high proportion of tasks perceived as illegitimate was also shown to be associated with role conflicts and high administrative workloads [12]. In Swedish primary care, a higher proportion of physicians (27%) perceived that they were required to perform illegitimate work tasks, particularly unnecessary work tasks, compared all other health care professionals [13]. Similar proportions of nurses and physicians (8% in both cases) perceived that they were required to perform many unreasonable work tasks. Although many work tasks are perceived as illegitimate, no study has reported which specific work tasks are considered illegitimate. Bridging this knowledge gap could provide valuable information about how to establish a sustainable work environment and ensure the efficient use of personnel.

### Aim

This study explored how physicians and nurses in Swedish primary care perceived the legitimacy of their work tasks and the use of their professional competence.

## Materials and methods

### Study design

This exploratory qualitative study collected data with semi-structured interviews of physicians and nurses. The data were evaluated with the manifest content analysis method, as described by Graneheim, Lindgren and Lundman [14].

### Settings

Sweden is divided into 21 healthcare regions. Primary care accounts for approximately 20% of all healthcare costs. In primary care, there is competition among public and private providers to register patients. Both public and private providers are allowed, when fulfilling advised requirements [15]. In Sweden, the average primary care staff consists of approximately 40% registered nurses, 21% physicians, 17% care administrators, 12% nurse assistants and 9% allied professionals (i.e. physiotherapists, occupational therapists, psychologists, counsellors, dieticians and chiropodists) [3]. The Region Östergötland, where the data were collected, is located in southern Sweden. This region has around 460,000 inhabitants [16]. There are approximately 1200 primary care centres in Sweden [15], and 44 are located Region Östergötland – eleven are privately operated on behalf of the region, and the remainder are publicly operated. The distributions of professionals are similar among all primary care staffs.

### Participants

We conducted strategic sampling to include both large and small primary care centres from urban, suburban and rural areas. The operation managers at 15 primary care centres were asked to approve participation in the study. Of these, nine agreed to allow the staff participate. Therefore, this study included four urban, two suburban and three rural centres. All nine primary care centres were publicly managed (i.e. no private primary care centres participated). In February 2020, these primary care centres served between 4900 and 21,065 patients (mean: 12,146). The mean numbers of residents served were approximately 14,600 in the urban primary care centres, 21,000 in the suburban centres and under 6000 in the rural centres.

After the operation managers of the primary care centres approved participation in the study, one investigator orally informed the staff physicians and nurses about the study. In most cases, the investigator informed the staff about the study during a workplace meeting; in a few cases, the operation managers provided the information to their staffs. Before the study started, the operation managers asked the physicians and nurses whether they were interested in participating in the study. All participants received an information letter about the study, which noted that the interviews would be audio-recorded, their input would be anonymised, and their confidentiality would be ensured. All participants signed a written informed consent form. The participants included 13 nurses (registered nurses) and 11 physicians (one resident and ten general practitioners). The characteristics of the study participants are presented in Table 1.

**Table 1.** Characteristics of primary care professionals that participated in the study.

Profession	*n*	Mean age years	Min–max	Women *n*	Men *n*	Years in profession (mean)	Years at workplace (mean)
Physicians	11	52.5	35–75	5	6	23^a^	10
Nurses	13	52.2	35–66	13	0	23	8

^a^Information missing about one participant.

### Data collection

The semi-structured interviews were conducted according to a study-specific interview guide with open-ended questions. All interview questions had been pilot-tested on two nurses before the study commenced. When the interviewing investigator contacted participants, they negotiated a time and place for conducting the interviews. Most interviews were performed at the participants’ workplace, but four interviews were performed over the telephone. The interviews lasted between 13 and 29 min, with an average of 21 min. The topic of the survey resulted in rather short interviews. However, the participants were given the opportunity to discuss any matters not covered in the survey.

The interviews were performed by the first author (EA). After an opening presentation of the project, the interview guide included questions about the physicians’ and nurses’ experiences. Overall, an open approach was applied; that is, the participants were encouraged to give free voice to their thoughts and experiences. At the conclusion of the interview, the participants were asked whether they wanted to add anything. The interviews were transcribed verbatim by a skilled external secretarial service. The data were collected between January 2020 and September 2020.

### Data analysis

The conventional content analysis method was previously described by Graneheim and Lundman [17]. Meaning units related to the aim of this study were identified, assigned to subcategories, and merged into categories, along with the original quotations. We read the data several times to avoid influence by preconceptions. The first author (EA) read all the interviews reflectively to form a naive understanding of the content. The second and third authors (MF and AS) read a majority of the interviews. After the initial reading, EA systematically worked through the material several times to gain a more in-depth familiarity with the content. Subsequently, all the investigators came to a consensus on the study material.

One of the authors was a physician, working part time in primary care and part time as a university lecturer. Another of the authors was a nurse with extensive experience working in primary care, but was also engaged in research at the time of the study. All authors read the data reflectively, with high awareness of any preconceptions. The authors found that the saturation of the data was sufficient.

### Ethical considerations

Upon seeking ethical approval for the study, the Swedish Ethical Review Authority determined that the study did not require its approval (Dnr. 2019-05600) [18].

## Results

The data were organised into three categories and eleven subcategories (Table 2).

**Table 2.** Categories for encoding the legitimacy of work tasks, the use of professional competence and features of the work situation, in primary care centres.

Categories	Subcategories
Work tasks	Collaboration between caregivers
	Problems with electronic information technology
	Unreasonable work tasks
	Legitimate work tasks
	Positive work tasks
Competence	Tasks in line with competence
	Unused competence
Demands in the work situation and potential improvements	Lack of resources
	Extensive workload
	Ambiguity about the appropriateness of work tasks
	Proposals for improvement

### Work tasks

#### Collaborations between caregivers

Primary-care staff had to collaborate in daily work with hospital-care staff and municipality personnel. Difficulties in these collaborations often led to discontentment. For example, physicians sometimes viewed referrals from hospital clinics as unnecessary or irrelevant. This perception often led to irritation, due to the inconvenience of dealing with tasks that the physician regarded as unnecessary; moreover, this attitude was often reflected in the physicians’ responses to the referring hospital clinics. Conversely, when physicians sent referrals to hospitals, they were often returned, which led to frustration. Moreover, physicians became irritated when hospital clinics requested results from several examinations before they would accept referrals from primary care. Sometimes, the primary-care physicians had little or no experience with some of the examinations the hospitals requested; thus, their involvement in those examinations were considered an unnecessary use of their time. Consequently, some physicians believed that hospital-clinic personnel sometimes viewed physicians and nurses more as secretaries than as healthcare professionals:

No, but it’s really that. We also get a lot of … they are referrals from the hospital, above all. In part, referrals where we have to … which I think are unnecessary; [like] referrals from the emergency ward [might want us] to do follow-ups that actually don’t need to be done. Or often, we might receive referrals from other clinics, where they think we need to write a referral for something, where they… [seem to think] that we should serve as a secretary, completely unnecessarily… but not at all …, just a poor use of time. (Physician 14)

Recently, primary care has been expected to treat very small children and patients with severe medical problems, such as complicated heart diseases. Some of these more difficult cases require competence, expertise and resources that are unavailable at primary care centres. Thus, treatments cannot be performed with an adequate level of patient safety:

There are many patients with multiple morbidities, and I feel like some clinics at the hospital have a hard time receiving patients and assisting them. That’s where it ends up in our lap, but we can’t … do anything more. We can’t help. We are not … I don’t have sufficient medical knowledge to help. That’s how I feel, and at the primary care centre, you try your best to help, and you try to send referrals, but you get them back all the time. (Physician 8)

#### Problems with electronic information technology

The electronic health record system was seen as problematic, but necessary. However, improvements generally took a very long time to implement:

Then, if you had a system that was … [and] worked like it should, that didn’t take two minutes for every step, then maybe it would be a bit easier. It is really frustrating to sit and try to help someone else, and then, the computer freezes and you can’t move and … yea. That’s my opinion. (Physician 11)

Double documentation (i.e. documenting the same information on more than one website or in different systems) was perceived as unnecessary. For example, nurses were required to report patient data in both the electronic health record and the Swedish National Diabetes Registry [19]. Although nurses had been told for several years that this double-entry problem would be solved, they continued to await a solution:

[…] And we have talked about this for many years, and at some point, it might get better, in that the national diabetes registry would be synchronised with Cosmic (electronic health records), but we’ll see. At least they are … they are trying. (Nurse 5)

Similarly, nurses had to document spirometry results in the spirometry programme and in the electronic health record, and then, they had to provide copies to physicians.

Both nurses and physicians were frustrated that the electronic health records of primary care centres and those of the municipalities were not connected or compatible; this lack of coordination led to extra work:

Yes, it really is … it is coordinating individual planning for the patient, … but when you … go to this link [Cosmic Link], it is … it has almost created a chat feature between the parties, between the home care, primary care centre, and inpatient [hospital] care. It almost becomes a chain. And you have to look at the colour-coded messages; [you have to know whether] you are [represented by] the red messages or orange or green or […] (Nurse 12)

#### Unreasonable work tasks

Nurses expressed distress over the lack of available appointments for patients to see physicians. Nurses had to allocate a considerable proportion of their work-time resolving this scheduling conundrum, and they perceived this work task as unreasonable:

Yes, [it would help if] I didn’t have to search for an [appointment] time. I have made my assessment. Someone else … either I would have a time [open for] booking … when I think that the patient … it still isn’t about whether the patient thinks they need it, but that I have determined that they need an [appointment] time, and if it isn’t possible, someone else should handle it. This seems very important. We have really worked on this [issue], because we have problems booking appointments… (Nurse 3)

In addition, the nurses perceived that it was unreasonable for them to have to call patients solely to deliver a message from a physician. Moreover, the nurses thought that it was unreasonable for them to perform, for example an electrocardiographic examination at the primary care centre, before a patient visited a cardiologist department. That is the nurses believed that an electrocardiographic examination should be performed at the cardiology clinic, during the patient’s visit. Another example of work tasks that nurses perceived as unreasonable was cleaning a physician’s office. Similarly, physicians found it unreasonable to be tasked with emptying dishwashers. Some physicians were frustrated, when they were required to perform tasks that kept them from using their professional expertise:

And it isn’t … about [the notion that] we are not the same as [other] people. We [physicians, nurses, and other staff] are valued the same as other people [are valued], but at some point, it actually [makes sense] that, with our expertise and with our salaries, we shouldn’t be used for non-specialised work. I mean, when I told others at RSG [the regional coordinating group] what we do … they couldn’t believe it was true; those are people that sit in higher positions in the region. (Physician 24)

Nurses were sometimes used for rather uncomplicated patient matters. However, rather than scheduling an appointment with nurse, some patients could be given advice on self-management over the phone.

Several participants described their administrative workload, such as documentation, as extensive. Similarly, some participants felt that much of the time that they spent on administrative tasks could have been better spent meeting with patients:

So, if you look at today, it’s … you have 30-minute appointments with the patients, for example; then, you have to document it all. And often … the patient comes in, and I try to get the appointment done in those 30 minutes, but you still have to complete the electronic health record. You have to try to get that done, and you have to send a message to the attending physician. So a lot of time is lost to administration: time that isn’t there. (Nurse 20)

#### Legitimate work tasks

The participants described patient meetings as meaningful, valuable and legitimate work. The broad variation of work tasks in primary care was also perceived as positive, and several participants found that the majority of their work tasks were legitimate:

Yes, they are. They are. It is reasonable for me to do this, because I think that’s what I should be doing. I should be meeting patients with multiple morbidities, I should be meeting with patients with metabolic illnesses, I should be helping people with chronic illnesses to live their lives as well as possible, I feel. I do not feel that someone else should be doing this. No, No. (Physician 23)

#### Positive work tasks

Nurses were responsible for documenting work errors and mistakes into appropriate administrative systems. The nurses found that this task was relevant and legitimate, because the work required the use of their medical competence. When a patient missed an appointment, that time gap could be spent on administrative duties, and nurses found that this arrangement encouraged good work flow. In addition, some administrative work tasks were considered positive:

Yes, I feel like … I have nothing against … I think it is rather nice to do administration. I am that kind of person, so I think it is fine. Often, it is administration I’ve created myself, because I have sent referrals, ordered X-rays, and other such things. (Physician 15)

### Competence

#### Tasks in line with competence

The staff felt that they largely performed tasks that they had been educated to perform, and that their work was linked to their competence and their profession:

I get to use my nursing expertise in many different ways. I think that’s very good. I have worked with different things, as an individual nurse, and I think that I have learned both purely medical knowledge − I feel like I get to use that, when I make medical assessments on the phone − and nursing skills … − so communication and conversations − and I also think that I get to use my skills by sharing them with others. (Nurse 3)

#### Unused competence

Nurses also noted that, sometimes, their competence could be used better. For example, they could be used in specific elder-care units led by nurses at the primary care centres. In addition, nurses believed that they could perform more patient assessments in the facility, rather than spending time on the telephone providing advice.

### Demands in the work situation and potential improvements

#### Lack of resources

The interviews revealed concerns about the shortage of staff, lack of time and room facilities, and a stressful work situation, in general. Some physicians wrote letters to patients, rather than dictating them, to ease the workload of busy care administrators. Primary care staff had to manage many patients that other clinics did not want or not had enough resources to manage. Moreover, in many cases, there was no other department that primary care staff could refer patients to:

And a lot has to do with … a lot I think … is frustrating with the care system; in general, no one wants to take responsibility for the patient. Instead, they pass the patient between departments and between different clinics. Where does the patient belong? And I sometimes feel a bit like primary care is [used as] a rubbish bin, in that way. Then, of course, we have to use specialist expertise, when it is really needed; but I think we need to improve primary care, if they [we in primary care] are going to take greater responsibility and be [treated as] the first line of care. (Nurse 13)

#### Extensive workload

The nurses expressed that their primary care centres were asked to perform more and more tasks without access to commensurate resources. This situation led to even more time spent on patient documentation, and the workload was difficult to manage during ordinary working hours.

Generally, physicians found that registering diagnoses in the electronic health record was very time consuming. This situation pressured them into looking for multiple diagnoses as a strategy for securing adequate financial reimbursement for the primary care centre, rather than providing any actual information value to the patient documentation.

For physicians, the requirements for preparing sick leave certificates had increased. Thus, they felt that this task took time away from other work and work with patients. Some physicians concluded that the work effort required to complete sick leave certificates was out of control. Moreover, the Swedish Social Insurance Agency frequently sent sick leave certificates back to the physicians with questions or demands to provide further information, which then generated even more work. In addition, patients were sometimes told by hospital clinics to contact the primary care centre for a sickness certificate, even when the hospital clinic provided care for the patient. One physician described the problem as follows:

My task is actually to try to assess … is this person sick? And, is it because of the illness that this person cannot work? And it’s almost impossible sometimes. An incredibly difficult task. But then if you think … yes, I judge that this person cannot work. Then I have to be some kind of lawyer and write or convince the insurance office in their language. And that is something … I personally feel is very difficult. And I know that it makes many of my colleagues furious […] (Physician 17)

Many nurses found that communicating with patients over the telephone after consulting with a physician was stressful. For example, some patients challenged the information that the nurses provided over the phone. This situation often resulted in the patients expressing their dissatisfaction with the care they received and generating more questions.

#### Ambiguity about the appropriateness of work tasks

Both nurses and physicians expressed ambiguous opinions about administrative tasks. Although they wanted more time to perform administrative tasks, they believed the more time they spent on administrative tasks, the less time they could spend with patients. Some physicians thought that primary care was administrative heavy, but they also thought that most of the administrative work needed to be performed. Nurses pointed out that they sometimes felt positive towards performing easy work tasks, like electrocardiographic examinations and removing sutures, which are often performed by nurse assistants. Some services were performed by nurse assistants at physicians’ offices, but not at nurses’ offices. Some nurses felt that they knew what was best for their situations, and therefore, they did not need nurse assistants to perform some tasks:

I have the opinion that now there are … two nurse assistants at this primary care centre, and they have a lot to do … they clean up … they empty things at the doctor’s [offices], but they don’t empty things for us. And they don’t refill supplies either … but, on the other hand, we are the ones that know what we need, so it’s completely fine, occasionally. (Nurse 12)

There were divergent opinions about who should manage the physicians’ individual waiting lists (i.e. patients listed with a specific physician). Some felt that handling their own lists could contribute to a sense of control over one’s work, and additionally, it provided variation in the workflow. Some nurses felt that managing administrative work left appallingly little time to meet with patients. Drop-in appointments at primary care centres were an alternative to scheduled appointments. However, some physicians found this system to be vulnerable and too inconsistent for the patients. For example, when a physician was on sick leave, the patient risked the situation of insufficient care continuity.

Ambiguous work tasks for primary care physicians included their many contacts with authorities; these tasks were seen as beneficial for patients, even though the work was not directly related to the patient’s healthcare.

#### Proposals for improvement

Several improvements were proposed. For example, the staff should handle their own schedules; patients should have the opportunity to report information about themselves digitally, *via* 1177 Vårdguiden, which is the Swedish website that allows patients to communicate digitally with caregivers (e.g. a primary care centre) [20]; and routine test results within normal range should automatically be approved in the electronic health record and not needed to be checked by a physician.

In addition, participants desired that more resources should be dedicated to giving advice over the phone. Nurses perceived that giving advice over the phone was difficult, and they wanted this work task to be performed by physicians. Physicians wanted resources to be redistributed from hospitals to primary care centres, to enlist primary care for managing basic care and to reserve hospital care for when it is really needed. The participants noted that these changes would require policy changes that would necessitate involving politicians.

## Discussion

This study on the perceptions of physicians and nurses in Swedish primary care regarding their work tasks showed that many frustrations were related to the perceived insufficient legitimacy of work tasks. Another perception was that primary care staff had a heavy workload. However, overall, the participants concluded that their competence was well used.

Physicians expressed great concern and discontentment over the poor interactions between primary care and hospital care staff. This issue seemed worrisome, occurred frequently, and was likely an important factor in workplace problems. This issue was also noted in a survey from Norway, which found that physicians reported that communication delays were hazardous to patient safety, because they increased the risk of adverse events [21]. In addition, Zuchowski et al. described continual communication challenges between primary care and specialty care; for example referral requests were often rejected [22]. Moreover, in our study, physicians felt that they often lacked the resources and hospital specialised knowledge needed to provide full service to their patients, and that these conditions ultimately compromised patient health. Furthermore, these conditions should be considered a negative influence on the healthcare staff involved, due to psychosocial stress.

Consistent with previous studies, we found that working with electronic health record systems was also considered challenging [23–25]. The importance of having useful electronic data systems cannot be over emphasised, because documentation in electronic health record systems plays a prominent role in primary care. Nurses had to document spirometry results in two electric health record systems; in addition, they had to report patient data in both the Swedish National Diabetes Registry and the electric health record system at the primary care centre. This redundancy might be frustrating and an inefficient use of time and competence. Törnqvist et al. concluded that the structure of electronic health records was a crucial factor in the ability to avoid this problem [26]. In this respect, the specific task of registering diagnoses in the electronic health record, to ensure a primary care centre received financial reimbursement, was identified as one example of a non-medical activity with low value, and therefore, it was considered illegitimate.

As described previously, the perception of work tasks as illegitimate may result in counterproductive behaviour. Indeed, the stress of illegitimate work tasks can negatively impact one’s professional identity [4], the work situation, in general and ultimately, the patient’s well-being.

The difficulty that nurses had in finding appointment times for patients that fit a physician’s schedule is a delicate problem. This problem highlighted the difficulties and the importance of dealing with the lack of resources in primary care. For example requiring nurses to clean physicians’ offices and requiring physicians to empty dishwashers seemed to be highly ineffective uses of medical resources.

However, despite the described negative comments, several positive opinions on the work situation were also expressed. For example, most staff considered that their individual competences were used effectively. According to ten Cate et al., competence is more than the possession of knowledge: it is also the ability to apply professional knowledge in clinical work to achieve optimal results [7]. The accurate use of individual competences is likely to benefit both the personnel involved and the quality and efficacy of the work. Thus, it is a better use of healthcare resources.

Participants in this study felt that they were overburdened with administrative work and lacked sufficient resources and time. For example, physicians believed that producing sickness certificates was an extremely demanding work task, consistent with findings in previous studies [27,28]. Consequently, this work task contributed to their work dissatisfaction. These adverse work conditions have been associated with poor quality of life among primary care staff members [29]. Therefore, it is highly important to allow healthcare workers to control their work situations, because it ensures that patients will receive the most effective and appropriate care possible [30]. Accordingly, allowing staff to manage their own schedules would be a positive, well-received policy. For example, Kjellström et al. emphasised the importance of professional autonomy among staff members [5], and Wright found that schedules developed without input from employees could lead to stress [31].

### Methodological considerations

Interviewing was considered an appropriate method for exploring perceptions of work tasks and how individual competence was used. The main strength of the study was that the content analyses of the interview data, a well-established method for qualitative studies [14,17], uncovered deep knowledge about how physicians and nurses perceived their work in primary care. The quality of the study performance was strengthened by the use of follow-up questions and by the fact that several investigators were involved in the analysis. This involvement resulted in a process of triangulation [32], which strengthened the credibility and authenticity of the study. Some value-charged words in the results might raise concern, but the wording was consistent with the participants’ expressions. Another strength of the study was that the primary care centres included were heterogeneous, in terms of geographic location and size.

This study also had a few limitations. Some interviews were conducted over the telephone. In addition, two of the authors worked in primary care, which might have introduced a risk of bias. However, Coar and Sim concluded that involving both clinicians and non-clinicians in data interpretations could offset some drawbacks [33]. On the other hand, there was a risk that the interviewers’ own feelings and opinions might have influenced the dialogue and the interpretation of the data. Another potential limitation was that the number of follow-up questions might not have been optimal. However, more questions would have somewhat prolonged the interviews. Nevertheless, the number of interviews was assessed as sufficient, and the data saturation was satisfactory. An alternative format could have been focus group interviews. However, focus groups might have attracted fewer participants, because participants would be required to attend a meeting outside their workplace and at a time that might have been inconvenient. Finally, our results might not be generalisable, because primary care differs considerably between countries (e.g. patient flow, workload, patient preferences and organisational factors). Additionally, the authenticity, dependability, confirmability and transferability of the results may be limited, from an international perspective.

## Conclusions

In primary care settings, strained work situations and perceptions of illegitimate work tasks may inflate discontentment and lead to negative work stress. Nevertheless, the participants of this study felt that their professional competences were used appropriately in the legitimate tasks performed in-between the illegitimate tasks.

## Clinical implications

The importance of sustainable working conditions and the thoughtful use of healthcare staff competence cannot be over emphasised. Medical competence, which is a product of many years of training and education, should be unhindered used to ensure that patients receive the best possible medical care. Further research is needed to explore how to optimise the use of physicians’ and nurses’ skills.

## Supplementary Material

Supplemental Material
