# Supplementary material for: ‘But there are so many referrals which are totally … only generating work and irritation’: a qualitative study of physicians’ and nurses’ experiences of work tasks in primary care in Sweden
Source: Scand J Prim Health Care. 2022 Nov 3;40(3):350–9. doi: 10.1080/02813432.2022.2139447 (PMC9848232; doi:10.1080/02813432.2022.2139447)
Supplement: Supplemental Material [file IPRI_A_2139447_SM6862.docx]

**Interview guide**

**Introduction**

- This study’s **purpose** is to describe how doctors and nurses experience the legitimacy of their work tasks. It is also to investigate how nurses and doctors experience the utilisation of their expertise in their daily work. Work tasks, in this case, are the tasks that you have at your workplace. **Legitimacy** of a task refers to tasks that you find meaningful and consider to be part of your professional role/profession at this workplace. By **illegitimate** is meant the opposite, i.e., those that you feel would not need to be done at all if things were better organised, i.e., **unnecessary**. **Unreasonable** is something that you think your profession should not have to do or you personally should not have to do.
- You have given **informed** **consent** to participate in the study but have the right to end your participation at any time without needing to provide an explanation.
- The information provided will be treated as confidential in accordance with **GDPR**.
- The interviews will be **recorded,** and you have the right to view the transcribed material.

**Interview questions**

- What is your profession?
- Do you have specialist training? If so, please specify what your specialist training is in.
- What is your year of birth?
- How long have you worked at this workplace?
- How long have you worked in your profession?
- Please describe a typical workday for you.
- How do you view your work tasks in relation to your professional competence?
- Patient-related tasks compared with non-patient-related tasks: what is included in each?
- How do you view your work tasks?
- How do you view your work tasks in terms of their legitimacy?
- Do you have tasks that you consider to be illegitimate? Expressed in another way, do you consider them outside your area of expertise?
- If so, what tasks do you consider unnecessary, that is to say, that do not need to be done at all?
- In what way are these tasks unnecessary?
- If so, what tasks do you consider unreasonable, that is to say, that should be performed by a different person/professional category?
- In what way are these tasks unreasonable?
- How do you view administrative tasks in your work?
- Describe your administrative tasks.
- Do you feel that you have administrative tasks that are unnecessary?
- If so, what are they?
- Do you feel that you have administrative tasks that are unreasonable?
- If so, what are they?
- How do you see a possible connection between the use of your expertise and the legitimacy of the work tasks (or illegitimacy if you like)?
- How would you like to organise your work so that you feel that you have time to complete your work tasks?
- Is there anything you would like to add?
